# The genome sequence of a soldier beetle, *Cantharis rustica *Fallén 1807

**DOI:** 10.12688/wellcomeopenres.17363.1

**Published:** 2021-11-26

**Authors:** Olga Sivell, Duncan Sivell, Natural History Museum Genome Acquisition Lab, Darwin Tree of Life Barcoding collective, Wellcome Sanger Institute Tree of Life programme, Wellcome Sanger Institute Scientific Operations: DNA Pipelines collective, Tree of Life Core Informatics collective, Darwin Tree of Life Consortium

**Affiliations:** 1 Department of Life Sciences, Natural History Museum, London, UK

**Keywords:** Cantharis rustica, sailor beetle, soldier beetle, genome sequence, chromosomal, Coleoptera

## Abstract

We present a genome assembly from an individual male *Cantharis rustica *(a soldier beetle; Arthropoda; Insecta; Coleoptera; Cantharidae). The genome sequence is 446 megabases in span. The majority (99.71%) of the assembly is scaffolded into 7 chromosomal pseudomolecules, with the X sex chromosome assembled.

## Species taxonomy

Eukaryota; Metazoa; Ecdysozoa; Arthropoda; Hexapoda; Insecta; Pterygota; Neoptera; Endopterygota; Coleoptera; Polyphaga; Elateriformia; Elateroidea; Cantharidae; Cantharinae; Cantharis; *Cantharis rustica* Fallén 1807 (NCBI:txid195172).

## Background

*Cantharis rustica* (Coleoptera, Cantharidae) is a soldier beetle that can be distinguished from other British soldier beetles by its black elytra, red pronotum with a black spot, and red (or partly red) femora with the remainder of the legs black in colour ( Fitton, 1973). It is common and widely distributed in southern Britain, but scarce and localised in the north ( Alexander, 2003; Alexander, 2014). The species prefers lowland grassland habitats, but also occurs in woodland and other habitats with tall grass. Adults can be found on vegetation and flower heads from mid-May till the end of June ( Alexander, 1991; Alexander, 2003; Fitton, 1973).

*Cantharis rustica* is predatory on invertebrates and has been observed to feed on a wide range of species, including *Sialis lutaria* (Neuroptera), *Malachius bipustulatus*, *Adalia bipunctata*, *Phyllobius* spp. (Coleoptera), *Tenthredopsis litterata*, *T. nassata*, *Arge gracilicornis* (Hymenoptera), *Empis livida*, *Bibio marci* and *Scatophaga stercoraria* (Diptera) ( Fincher, 1951; Hobby, 1932). The adults and larvae of soldier beetles are mainly carnivorous, feeding on live and dead invertebrates, but will also feed on plant material ( Alexander, 1991). The cantharid larvae have a velvety appearance and can be found in leaf litter and in the top layers of soil ( Alexander, 1991; Fitton, 1973).

The karyotype of *Cantharis rustica* has been described and illustrated by James & Angus (2007); males have an X0 sex chromosome system. The high-quality genome sequence described here is, to our knowledge, the first one reported for *Cantharis rustica* and has been generated as part of the Darwin Tree of Life project. It will aid in understanding the biology, physiology and ecology of the species.

## Genome sequence report

The genome was sequenced from one male *C. rustica* collected from Wigmore Park, Luton, UK (latitude 51.88378, longitude -0.36861422). A total of 43-fold coverage in Pacific Biosciences single-molecule long reads and 48-fold coverage in 10X Genomics read clouds were generated. Primary assembly contigs were scaffolded with chromosome conformation Hi-C data. Manual assembly curation corrected 60 missing/misjoins and removed 7 haplotypic duplications, reducing the assembly length by 0.75% and the scaffold number by 72.13%, and increasing the scaffold N50 by 133.18%.

**Figure 1.**
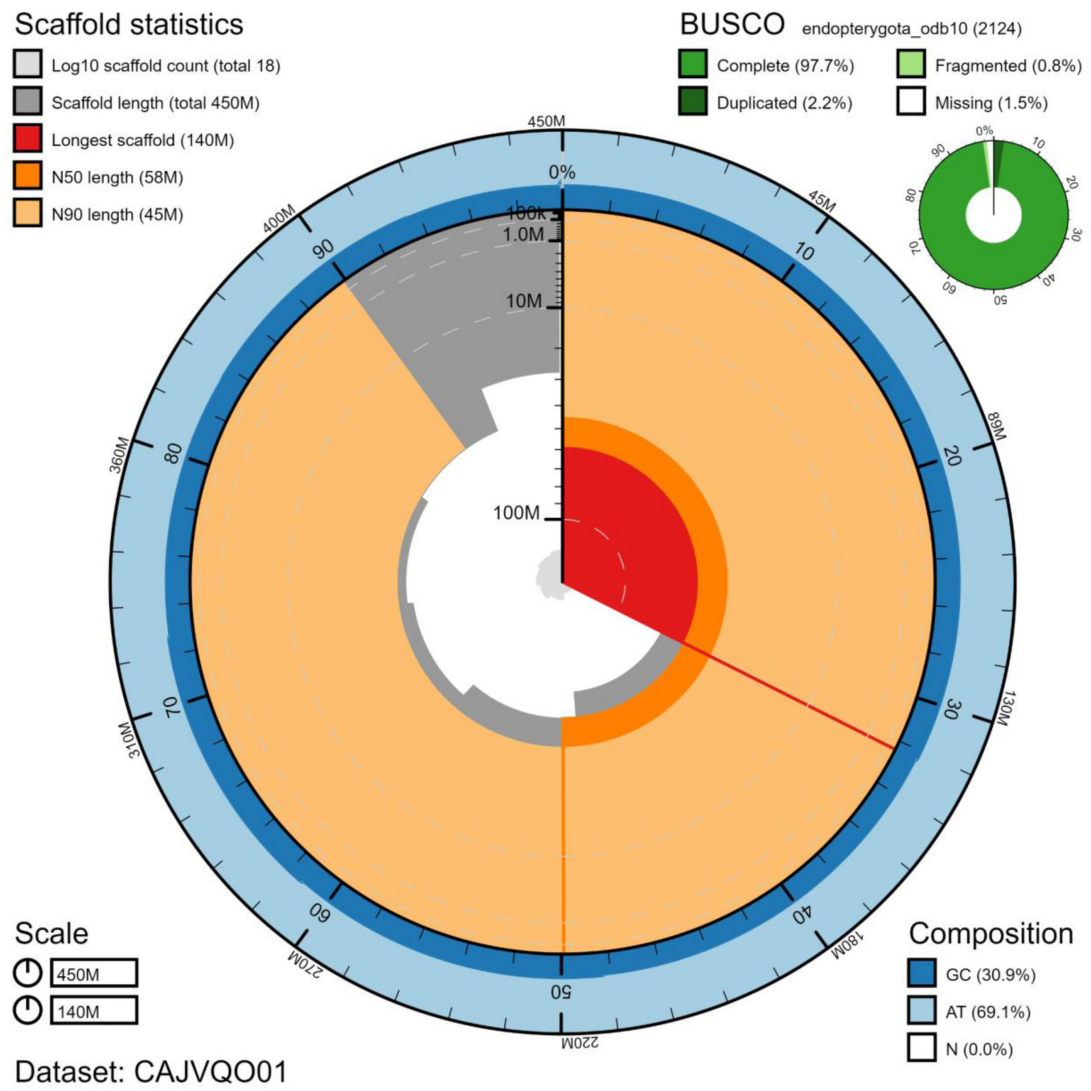
Genome assembly of *Cantharis rustica*, icCanRust1.1: metrics. The main plot is divided into 1,000 size-ordered bins around the circumference with each bin representing 0.1% of the 445,936,792 bp assembly. The distribution of scaffold lengths is shown in dark grey with the plot radius scaled to the longest scaffold present in the assembly (144,510,368 bp, shown in red). Orange and pale-orange arcs show the N50 and N90 scaffold lengths (57,811,309 and 44,785,338 bp), respectively. The pale grey spiral shows the cumulative scaffold count on a log scale with white scale lines showing successive orders of magnitude. The blue and pale-blue area around the outside of the plot shows the distribution of GC, AT and N percentages in the same bins as the inner plot. A summary of complete, fragmented, duplicated and missing BUSCO genes in the endopterygota_odb10 set is shown in the top right. An interactive version of this figure is available at https://blobtoolkit.genomehubs.org/view/icCanRust1.1/dataset/CAJVQO01/snail.

The final assembly has a total length of 446 Mb in 17 sequence scaffolds with a scaffold N50 of 57.8 Mb ( Table 1). The majority, 99.71%, of the assembly sequence was assigned to 7 chromosomal-level scaffolds, representing 6 autosomes (numbered by sequence length), and the X sex chromosome ( Figure 1– Figure 4; Table 2). The assembly has a BUSCO v5.1.2 ( Manni *et al*., 2021) completeness of 97.7% (single 95.5%, duplicated 2.2%) using the endopterygota_odb10 reference set. While not fully phased, the assembly deposited is of one haplotype. Contigs corresponding to the second haplotype have also been deposited.

**Table 1.** Genome data for *Cantharis rustica*, icCanRust1.1.

*Project accession data*	
Assembly identifier	icCanRust1.1
Species	*Cantharis rustica*
Specimen	icCanRust1
NCBI taxonomy ID	NCBI:txid195172
BioProject	PRJEB45190
BioSample ID	SAMEA7524272
Isolate information	Male, thorax (genome assembly), abdomen (Hi-C)
*Raw data accessions*	
PacificBiosciences SEQUEL II	ERR6558187
10X Genomics Illumina	ERR6054935-ERR6054938
Hi-C Illumina	ERR6054939
*Genome assembly*	
Assembly accession	GCA_911387805.1
Accession of alternate haplotype	GCA_911387815.1
Span (Mb)	446
Number of contigs	75
Contig N50 length (Mb)	17.8
Number of scaffolds	17
Scaffold N50 length (Mb)	57.8
Longest scaffold (Mb)	144.5
BUSCO * genome score	C:97.7%[S:95.5%,D:2.2%], F:0.8%,M:1.5%,n:2124

*BUSCO scores based on the endopterygota_odb10 BUSCO set using v5.1.2. C= complete [S= single copy, D=duplicated], F=fragmented, M=missing, n=number of orthologues in comparison. A full set of BUSCO scores is available at https://blobtoolkit.genomehubs.org/view/icCanRust1.1/dataset/CAJVQO01/busco.

**Figure 2.**
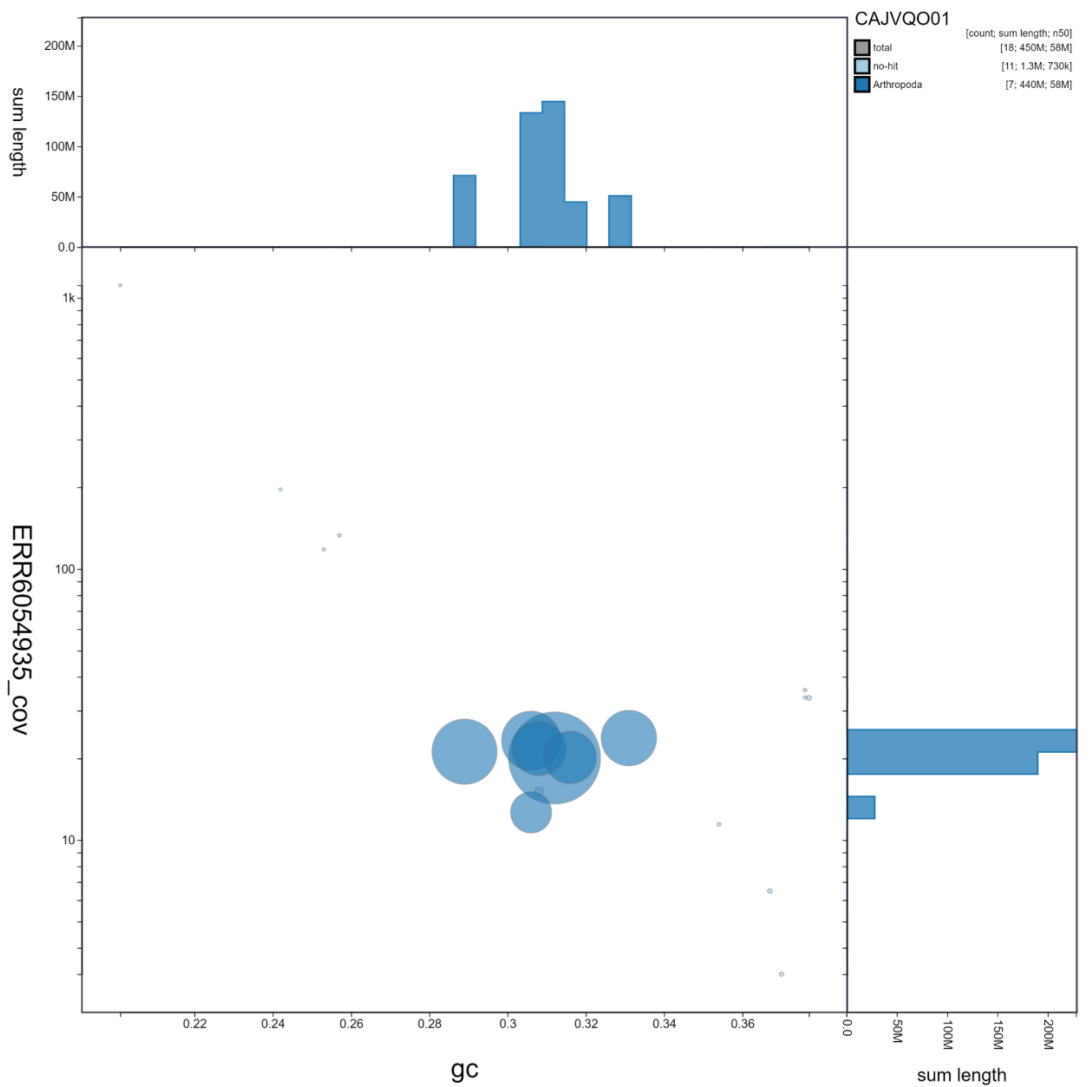
Genome assembly of *Cantharis rustica*, icCanRust1.1: GC coverage. BlobToolKit GC-coverage plot. Scaffolds are coloured by phylum. Circles are sized in proportion to scaffold length Histograms show the distribution of scaffold length sum along each axis. An interactive version of this figure is available at https://blobtoolkit.genomehubs.org/view/icCanRust1.1/dataset/CAJVQO01/blob.

**Figure 3.**
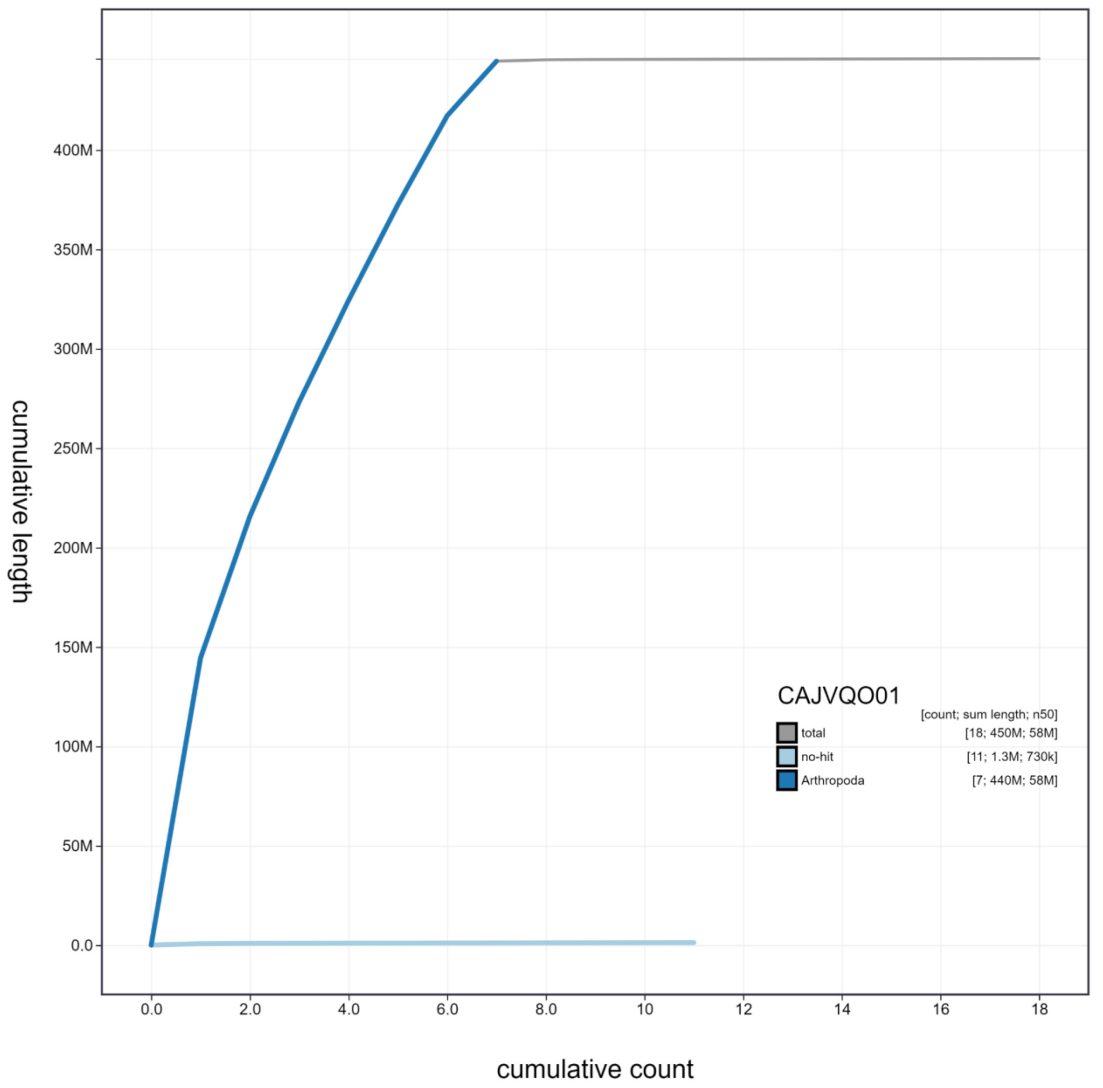
Genome assembly of *Cantharis rustica*, icCanRust1.1: cumulative sequence. BlobToolKit cumulative sequence plot. The grey line shows cumulative length for all scaffolds. Coloured lines show cumulative lengths of scaffolds assigned to each phylum using the buscogenes taxrule. An interactive version of this figure is available at https://blobtoolkit.genomehubs.org/view/icCanRust1.1/dataset/CAJVQO01/cumulative.

**Figure 4.**
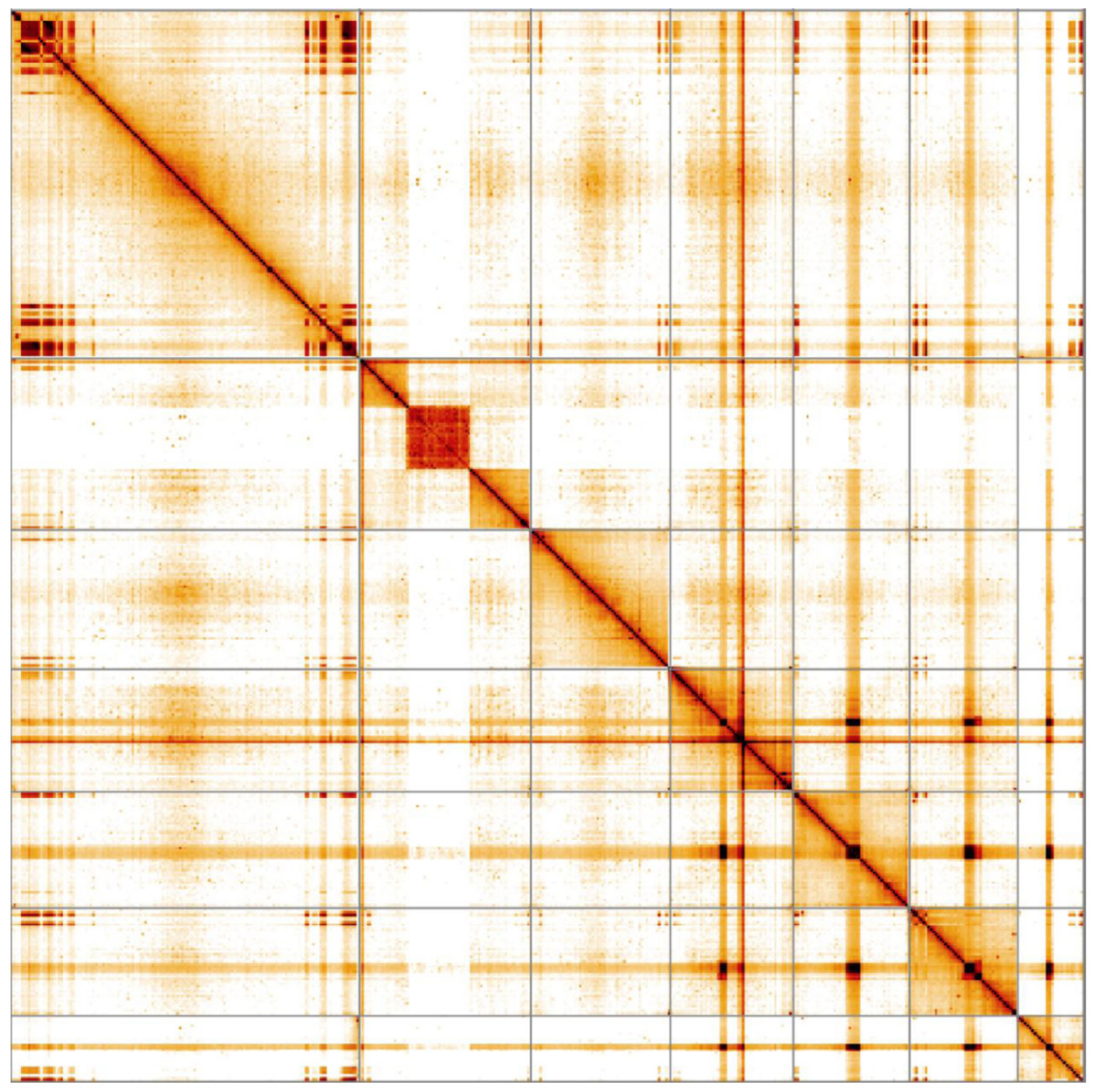
Genome assembly of *Cantharis rustica*, icCanRust1.1: Hi-C contact map. Hi-C contact map of the icCanRust1.1 assembly, visualised in HiGlass.

**Table 2.** Chromosomal pseudomolecules in the genome assembly of Rhagonycha fulva, icRhaFulv1.1.

INSDC accession	Chromosome	Size (Mb)	GC%
OU426877.1	1	144.51	31.2
OU426878.1	2	71.07	28.9
OU426879.1	3	57.81	30.6
OU426880.1	4	50.92	33.1
OU426881.1	5	48.21	30.8
OU426882.1	6	44.79	31.6
OU426883.1	X	27.33	30.6
OU426884.1	MT	0.02	20.3
-	Unplaced	1.29	32.7

Chromosome 2 contains a large heterochromatic region of low confidence at approximately 20-46 Mb. This block consists of a number of scaffolds with high repeat content that can be localised to chromosome 2 but their order and orientation with respect to each other is unsure. Large islands of similar tandem repeat with high GC content are observed near both poles of Chromosome 1. Small islands of a related repeat are observed in all other chromosomes.

## Methods

### Sample acquisition and DNA extraction

A single female *C. rustica* (icCanRust1) was collected from Wigmore Park, Luton, UK (latitude 51.88378, longitude -0.36861422) by Olga Sivell, Natural History Museum, using a net. The sample was identified by Duncan Sivell, Natural History Museum and snap-frozen on dry ice. Unfortunately, as this specimen was collected during a COVID-19 lockdown, no image was captured prior to preservation.

DNA was extracted at the Tree of Life laboratory, Wellcome Sanger Institute. The icCanRust1 sample was weighed and dissected on dry ice with tissue set aside for Hi-C sequencing. Thorax tissue was disrupted using a Nippi Powermasher fitted with a BioMasher pestle. Fragment size analysis of 0.01-0.5 ng of DNA was then performed using an Agilent FemtoPulse. High molecular weight (HMW) DNA was extracted using the Qiagen MagAttract HMW DNA extraction kit. Low molecular weight DNA was removed from a 200-ng aliquot of extracted DNA using 0.8X AMpure XP purification kit prior to 10X Chromium sequencing; a minimum of 50 ng DNA was submitted for 10X sequencing. HMW DNA was sheared into an average fragment size between 12-20 kb in a Megaruptor 3 system with speed setting 30. Sheared DNA was purified by solid-phase reversible immobilisation using AMPure PB beads with a 1.8X ratio of beads to sample to remove the shorter fragments and concentrate the DNA sample. The concentration of the sheared and purified DNA was assessed using a Nanodrop spectrophotometer and Qubit Fluorometer and Qubit dsDNA High Sensitivity Assay kit. Fragment size distribution was evaluated by running the sample on the FemtoPulse system.

### Sequencing

Pacific Biosciences HiFi circular consensus and 10X Genomics read cloud DNA sequencing libraries were constructed according to the manufacturers’ instructions. Sequencing was performed by the Scientific Operations core at the Wellcome Sanger Instituteon Pacific Biosciences SEQUEL II and Illumina HiSeq X instruments. Hi-C data were generated from abdomen tissue using the Arima Hi-C+ kit and sequenced on an Illumina NovaSeq 6000 instrument.

### Genome assembly

Assembly was carried out with Hifiasm ( Cheng *et al*., 2021); haplotypic duplication was identified and removed with purge_dups ( Guan *et al*., 2020). One round of polishing was performed by aligning 10X Genomics read data to the assembly with longranger align, calling variants with freebayes ( Garrison & Marth, 2012). The assembly was then scaffolded with Hi-C data ( Rao *et al*., 2014) using SALSA2 ( Ghurye *et al*., 2019). The assembly was checked for contamination and corrected using the gEVAL system ( Chow *et al*., 2016) as described previously ( Howe *et al*., 2021). Manual curation was performed using gEVAL, HiGlass ( Kerpedjiev *et al*., 2018) and Pretext. The mitochondrial genome was assembled using MitoHiFi ( Uliano-Silva *et al*., 2021). The genome was analysed and BUSCO scores generated within the BlobToolKit environment ( Challis *et al*., 2020). Table 3 contains a list of all software tool versions used, where appropriate.

**Table 3.** Software tools used.

Software tool	Version	Source
Hifiasm	0.12-r304	Cheng *et al*., 2021
purge_dups	1.2.3	Guan *et al*., 2020
SALSA2	2.2	Ghurye *et al*., 2019
longranger align	2.2.2	https://support.10xgenomics. com/genome-exome/software/ pipelines/latest/advanced/other- pipelines
freebayes	1.3.1-17- gaa2ace8	Garrison & Marth, 2012
MitoHiFi	2.1	Uliano-Silva *et al*., 2021
gEVAL	N/A	Chow *et al*., 2016
HiGlass	1.11.6	Kerpedjiev *et al*., 2018
PretextView	0.2.x	https://github.com/wtsi-hpag/ PretextView
BlobToolKit	2.6.2	Challis *et al*., 2020

### Ethics/compliance issues

The materials that have contributed to this genome note have been supplied by a Darwin Tree of Life Partner. The submission of materials by a Darwin Tree of Life Partner is subject to the Darwin Tree of Life Project Sampling Code of Practice. By agreeing with and signing up to the Sampling Code of Practice, the Darwin Tree of Life Partner agrees they will meet the legal and ethical requirements and standards set out within this document in respect of all samples acquired for, and supplied to, the Darwin Tree of Life Project. Each transfer of samples is further undertaken according to a Research Collaboration Agreement or Material Transfer Agreement entered into by the Darwin Tree of Life Partner, Genome Research Limited (operating as the Wellcome Sanger Institute), and in some circumstances other Darwin Tree of Life collaborators.

## Data availability

European Nucleotide Archive: Cantharis rustica (sailor beetle). Accession number PRJEB45190; https://identifiers.org/ena.embl/PRJEB45190.

The genome sequence is released openly for reuse. The *C. rustica* genome sequencing initiative is part of the Darwin Tree of Life (DToL) project. All raw sequence data and the assembly have been deposited in INSDC databases.The genome will be annotated using the RNA-Seq data and presented through the Ensembl pipeline at the European Bioinformatics Institute. Raw data and assembly accession identifiers are reported in Table 1.

## Author information

Members of the Natural History Museum Genome Acquisition Lab are listed here: https://doi.org/10.5281/zenodo.4790043.

Members of the Darwin Tree of Life Barcoding collective are listed here: https://doi.org/10.5281/zenodo.4893704.

Members of the Wellcome Sanger Institute Tree of Life programme collective are listed here: https://doi.org/10.5281/zenodo.5377053.

Members of Wellcome Sanger Institute Scientific Operations: DNA Pipelines collective are listed here: https://doi.org/10.5281/zenodo.4790456.

Members of the Tree of Life Core Informatics collective are listed here: https://doi.org10.5281/zenodo.5013542.

Members of the Darwin Tree of Life Consortium are listed here: https://doi.org/10.5281/zenodo.4783559.
