# Supplementary material for: The genome sequence of a soldier beetle, Cantharis rustica Fallén 1807
Source: Wellcome Open Res. 2021 Nov 26;6:325. [Version 1] doi: 10.12688/wellcomeopenres.17363.1 (PMC9975405; doi:10.12688/wellcomeopenres.17363.1)
Supplement: Peer review history

# Peer review: Reviewer response for version 1

**DOI:** 10.21956/wellcomeopenres.19198.r53801

**Published:** 2023-01-09

**Authors:** Sean Schoville

**Affiliations:** 1 Department of Entomology, University of Wisconsin-Madison, Madison, WI, USA

The authors describe the first genome assembly of the soldier beetle *Cantharis rustica*. Combining HiFi long reads and HI-C data from an adult male beetle, a chromosomal-level assembly of ~446 Mb is produced. The genome contains six autosomes and one X chromosome (the species is XO). Several metrics suggest that genome has a high completeness and low contamination.

 There is a discrepancy in the paper as to whether this represents a male or female (line 1 in Methods says female, the abstract says male). If uncertain, perhaps examining read coverage on the X is useful. To this point, it is not clear in the text how the X has been identified.

 The program "purge_dups" was used on the hifiasm assembly, although hifiasm has a similar function embedded. It may be worth reporting the change in size or other metrics associated with the additional purge, as you may be removing real genome duplication.

 Could you report on heterozygosity of the genome? It is unclear what information Freebayes has added to your assembly otherwise.

 A short note on the mitochondrial genome (is it complete, same gene order as canonical Coleoptera) would be helpful.

 Minor revisions:

- Abstract: The last sentence makes it unclear whether the X chromosome is one of the seven pseudomolecules or not. I would rephrase.
- On page 3- I think you don't need the qualifier "to our knowledge" as it is undoubtedly the first given a scan of the literature.
- On Page 3, reference to "the X sex chromsome" could be more usefully written as "the single sex chromosome (XO)".
- One Page 3, this sentence is vague "Small islands of a related repeat are observed in all other chromosomes." Is the repeat a conserved motif found repeatedly, and is it the same as the tandem repeat referred to in the preceding sentence?
- Page 7, reference to "Manual curation" should be clarified as curation of the assembly, not of a gene set.
- Page 8, remove "the" before RNA-seq data

Are sufficient details of methods and materials provided to allow replication by others?

Yes

Is the rationale for creating the dataset(s) clearly described?

Yes

Are the datasets clearly presented in a useable and accessible format?

Yes

Are the protocols appropriate and is the work technically sound?

Yes

Reviewer Expertise:

Evolutionary genomics of insects.

I confirm that I have read this submission and believe that I have an appropriate level of expertise to confirm that it is of an acceptable scientific standard.

# Peer review: Reviewer response for version 1

**DOI:** 10.21956/wellcomeopenres.19198.r49746

**Published:** 2022-04-11

**Authors:** Ardha Apriyanto

**Affiliations:** 1 Research and Development, PT. Astra Agro Lestari Tbk, Jakarta Timur, Indonesia; 2 Biopolymer Analytics, University of Potsdam, Potsdam-Golm, Germany

In this report, the authors present a genome sequence of *Cantharis rustica *(Coleoptera:Cantharidae), a soldier beetle. The sequence reported is novel and contains exciting information. Also, the dataset will provide a valuable resource for the scientific community. The genome reported in this study utilized a hybrid assembly using short and long reads. As a result, it produces a very high-quality genome assembly. However, this report does not cover the annotation of the genome sequence aided by RNAseq.

 Regarding the manuscript, I found some minor mistakes that need revision:

- The species' name in table 2 should be *Cantharis rustica *(not *Rhagonycha fulva*).
- Also, the dataset name should be icCanRust1.1 (not icRhaFulv1.1).
- All species names should be italic, for example, Cantharis rustica, in the data availability section *.*

In summary, the way of research reporting is very similar to its sister reports I found in this journal, which lacks variation. However, I understand that this report is part of a large genome project consortium that commonly uses a similar assembly pipeline and reporting style. I would like to recommend it for indexing with minor revision.

Are sufficient details of methods and materials provided to allow replication by others?

Yes

Is the rationale for creating the dataset(s) clearly described?

Yes

Are the datasets clearly presented in a useable and accessible format?

Yes

Are the protocols appropriate and is the work technically sound?

Yes

Reviewer Expertise:

Genome assembly, Genomics, Transcriptomics, Bioinformatics

I confirm that I have read this submission and believe that I have an appropriate level of expertise to confirm that it is of an acceptable scientific standard.
